# Analysis of the interaction of Plexin-B1 and Plexin-B2 with Rnd family proteins

**DOI:** 10.1371/journal.pone.0185899

**Published:** 2017-10-17

**Authors:** Thomas Wylie, Ritu Garg, Anne J. Ridley, Maria R. Conte

**Affiliations:** Randall Division of Cell and Molecular Biophysics, King’s College London, Guy’s Campus, London, United Kingdom; Russian Academy of Medical Sciences, RUSSIAN FEDERATION

## Abstract

The Rnd family of proteins, Rnd1, Rnd2 and Rnd3, are atypical Rho family GTPases, which bind to but do not hydrolyse GTP. They interact with plexins, which are receptors for semaphorins, and are hypothesised to regulate plexin signalling. We recently showed that each Rnd protein has a distinct profile of interaction with three plexins, Plexin-B1, Plexin-B2 and Plexin-B3, in mammalian cells, although it is unclear which region(s) of these plexins contribute to this specificity. Here we characterise the binary interactions of the Rnd proteins with the Rho-binding domain (RBD) of Plexin-B1 and Plexin-B2 using biophysical approaches. Isothermal titration calorimetry (ITC) experiments for each of the Rnd proteins with Plexin-B1-RBD and Plexin-B2-RBD showed similar association constants for all six interactions, although Rnd1 displayed a small preference for Plexin-B1-RBD and Rnd3 for Plexin-B2-RBD. Furthermore, mutagenic analysis of Rnd3 suggested similarities in its interaction with both Plexin-B1-RBD and Plexin-B2-RBD. These results suggest that Rnd proteins do not have a clear-cut specificity for different Plexin-B-RBDs, possibly implying the contribution of additional regions of Plexin-B proteins in conferring functional substrate selection.

## Introduction

Plexins are a family of transmembrane cell surface receptors [1] which are divided into four families based on structural criteria, Plexins A, B, C and D [2,3]. The cytoplasmic region of plexins is highly conserved, consisting mainly of a bipartite segment with high homology to the GTPase-activating domain (GAP domain) found in proteins that act as GAPs for Ras family proteins, such as p120RasGAP [4]. RasGAP domains stimulated hydrolysis of GTP bound to Ras family proteins such as Ras, Rap and R-Ras, thereby converting them to their inactive GDP-bound conformation. The two regions in Plexins that together form the RasGAP domain are separated by approximately 200 residues, and a ~120 residue region within this forms an independent folding unit that binds to several Rho family GTPases, termed the Rho-binding domain (RBD) [5,6].

Of the Plexin-B family, Plexin-B1 is the best studied, primarily transducing signals from the semaphorin Sema4D and with several roles in development and disease [7–9]. Plexins in neurons are involved in neurite retraction and contribute to neuronal guidance *in vivo*. This is mediated by their GAP activity on R-Ras and/or Rap1 [10–13]. Whether Plexin-B2 or Plexin-B3 act as GAPs for Rap1 or R-Ras is not known, although we showed that all three Plexin-B proteins interact with R-Ras and Rap1 [14]. Plexin-B family members contribute to cancer progression, for example Sema4D induces invasive growth of cancer cells by signalling through Plexin-B1, which associates with and activates the tyrosine kinase receptors Met or Ron [15,16]. Similarly, the Plexin-B3 ligand Sema5A transactivates Met [17] and Plexin-B2 synergises with Met to induce glioma cell invasion [18]. Plexin-B1 can also transactivate the tyrosine kinase receptor ErbB2 in breast cancer cells to stimulate cell migration [19].

Several of the functions of Plexin-B proteins are linked to their ability to interact with Rho family GTPases. Most Rho GTPases cycle between an active GTP-bound conformation and an inactive GDP-bound conformation [20]. When bound to GTP they signal through their downstream targets. The atypical Rho family members are characterised by a lack of intrinsic GTP hydrolysis and include Rnd1, Rnd2 and Rnd3 [20–22]. The proteins were named Rnd because they induce cell rounding when overexpressed in cultured cells in vitro, due to a decrease in actin stress fibres and integrin-mediated focal adhesions. Rnd1 has been found to be required for Plexin-B1 R-RasGAP activity [12,23], whilst Rnd3 binding to Plexin-B2 stimulates RhoA activity in cortical neuron migration [24]. Rnd3 interaction with Plexin-B2 also inhibits cancer cell invasion [14].

Given that many of the Plexins A-D interact with a number of Rho GTPases, the current working hypothesis is that different Rho GTPases have distinct roles in Plexin signalling and, in support of this view, different binding affinities of Plexin family members for small Rho GTPases have been described [25].

Quantitative analyses of the interaction of Rnd proteins with Plexin-B1 have been reported previously with conflicting results (S1 Table). In one study, isothermal titration calorimetry (ITC) data revealed that Rnd1 but not Rnd2 associated with Plexin-B1-RBD, although no difference in binding affinity was observed by surface plasmon resonance (SPR) analysis conducted in parallel [26]. On the other hand, in a different study, the association of Plexin-B1-RBD with the 3 Rnd proteins was found to be similar by ITC [6]. No biophysical data characterising the binary interaction of Plexin-B2 RBD with Rnd proteins has been reported so far. However, co-immunoprecipitation experiments with full length Plexin-B1 and Plexin-B2 showed that Rnd2 was able to interact with both Plexin-B1 and Plexin-B2 whilst Rnd3 interacted preferentially with Plexin-B2 and much more weakly with Plexin-B1 [14,24].

Here, we have carried out a direct comparison of the molecular interactions of Rnd1, Rnd2 and Rnd3 to Plexin-B1-RBD and Plexin-B2-RBD using biophysical techniques. We do not observe any striking difference in binding affinity between any Rnd protein and Plexin-B1-RBD or Plexin-B2-RBD, although Rnd1 binds the strongest to Plexin-B1 and weakest to Plexin-B2. Analysis of mutants located in the predicted interface of Rnd3 with Plexin-B2 also suggests similarities in molecular recognition between Rnd3 and the two Plexin-B RBDs.

## Materials and methods

### Protein expression and purification

The regions encoding the RBD of Plexin-B1 (residues 1746–1851) and Plexin-B2 (1452–1562) and the GTP-binding domains of Rnd1 (1–191), Rnd2 (2–184) and Rnd3 (16–200) were amplified by PCR and subcloned into pETDuet-1 for expression in *E*. *coli*. The proteins contain an N-terminal hexahistidine tag to aid purification. A Tobacco Etch Virus (TEV) Protease cleavage site was engineered to enable removal of the tag after purification. Proteins were overexpressed in *E*. *coli* overnight at 18°C by addition of 1 mM isopropyl 1-thio-β-D1-galactopyranoside (IPTG). Rnd1 was expressed in *E*. *coli* Rosetta 2 (DE3), while Plexin-B1-RBD, Plexin-B2-RBD, Rnd2 and Rnd3 were expressed in *E*. *coli* BL21 (DE3). Proteins were purified using Nickel-NTA chromatography, followed by incubation with TEV Protease at a 1:50 molar ratio to remove the hexahistidine-tags, with the exception of Plexin-B1-RBD, for which removal of the tag was inefficient. The purity of the proteins was assessed using SDS-PAGE, and in some cases either ion exchange or size exclusion chromatography was used to obtain the level of purity required for biophysical experiments. For ion affinity chromatography, samples were loaded onto a HiTrap diethylaminoethanol (DEAE) FF 5 mL column (GE Healthcare) in 20 mM Tris pH 7.5, 5 mM MgCl_2_ and 1 mM Dithiothreitol (DTT), then eluted using a salt concentration gradient from 0 to 1 M KCl over 40 column volumes. A Superdex75 10/300 column (GE Healthcare) was used for size exclusion chromatography following the same procedure as described below for analytical gel filtration.

The five Rnd3 point mutations have been described previously [14]. They were cloned into pETDuet-1, and the proteins purified in the same way as wild type Rnd3, with the exception of Rnd3-V87R and Rnd3-F124Y which were expressed in *E*. *coli* Rosetta 2 (DE3).

^1^H NMR was used to assess the folding state of the purified proteins (data not shown). All proteins were dialysed in the appropriate ITC buffer (see below) and flash frozen.

The Rnd proteins co-purified bound to GTP [5,27] as confirmed by their UV absorbance profile at 254/280 nm [28]. Protein concentrations were calculated based upon the near-UV absorption (ε280) using theoretical extinction coefficients derived from ExPASy [29]; for Rnd proteins, to take into account the presence of GTP, the extinction coefficient for guanine nucleotides at 280 nm, 7765 cm^–1^*M*^–1^, was added to the extinction coefficient of the protein calculated with ExPASy [28].

### Isothermal titration calorimetry

Interaction studies between Rnd proteins and the RBDs of Plexin-B1 and Plexin-B2 were carried out at 25°C using a MicroCal Isothermal Titration Calorimeter ITC200 instrument (Malvern). For most experiments the proteins were prepared in either 50 mM sodium phosphate pH 7.0, 50 mM NaCl, 4 mM MgCl_2_ and 3 mM DTT (buffer 1) or 50 mM sodium phosphate pH 7.0, 150 mM NaCl, 4 mM MgCl_2_ and 3 mM DTT (buffer 2). The experiments were conducted following standard procedures as described previously [30]. Typically, a 40 μL solution of either Plexin-B1-RBD or Plexin-B2-RBD at a concentration of 400–600 μM was placed in the syringe and titrated into a 330 μL solution of Rnd1, Rnd2 or Rnd3 protein at a concentration of 40–60 μM in the cell. The ITC experiment typically consisted of twenty injections of 2 μL each with a spacing of 180 seconds between injections. Heat produced by titrant dilution was obtained by a control experiment, titrating into buffer alone, under the same conditions. This was verified to be negligible for Plexin-B2-RBD, but not for Plexin-B1-RBD, where a dimer dissociation curve was observed ([5] and see below). The MicroCal-Origin 7.0 software package was used to fit the integrated heat data obtained for the titrations corrected for heats of dilution, using a non-linear least-squares minimization algorithm based on an independent binding sites model. ΔH (reaction enthalpy change in kcal/mol), Ka (equilibrium association constant per molar) and n (molar ratio of the proteins in the complex) were the fitting parameters. The reaction entropy was calculated using the relationships ΔG = ΔH-TΔS = -RTlnKa.

### Analytical size exclusion chromatography

Size exclusion chromatography was performed at 4°C on a Superdex75 10/300 (GE Healthcare) equilibrated with 50 mM sodium phosphate pH 7.0, 150 mM NaCl, 4 mM MgCl_2_ and 3 mM DTT. A 500 μL sample containing 100 μM of each protein in the same buffer was loaded onto the column and run through at a rate of 0.5 ml/min. Elution of the proteins from the column was tracked by absorbance at 280 nm of the elute. Column calibration for molecular weight estimation was performed as described previously [30].

## Results

### Analysis of Plexin-B1-RBD interactions by isothermal titration calorimetry

We investigated the binding affinities of Plexin-B1-RBD for Rnd1, Rnd2 and Rnd3 using ITC. In initial experiments, we used similar buffer conditions as described previously for Plexin-B1-RBD with Rnd1 [26] (50 mM NaCl–buffer 1; S1 Fig, S2 Table), and as expected for the Plexin-B1-Rnd1 interaction the thermodynamic profile obtained matched closely with what was previously reported [26]. The association of Rnd2 with Plexin-B1-RBD could not be fitted to a single binding isotherm under these experimental conditions however, mainly because the ITC data were affected by significant heat contributions from non-specific binding events (S1 Fig) [31,32]. Notably, no ITC data for Plexin-B1-Rnd2 interaction were shown previously [26], although an estimated Kd >20 μM was reported.

**Fig 1:**
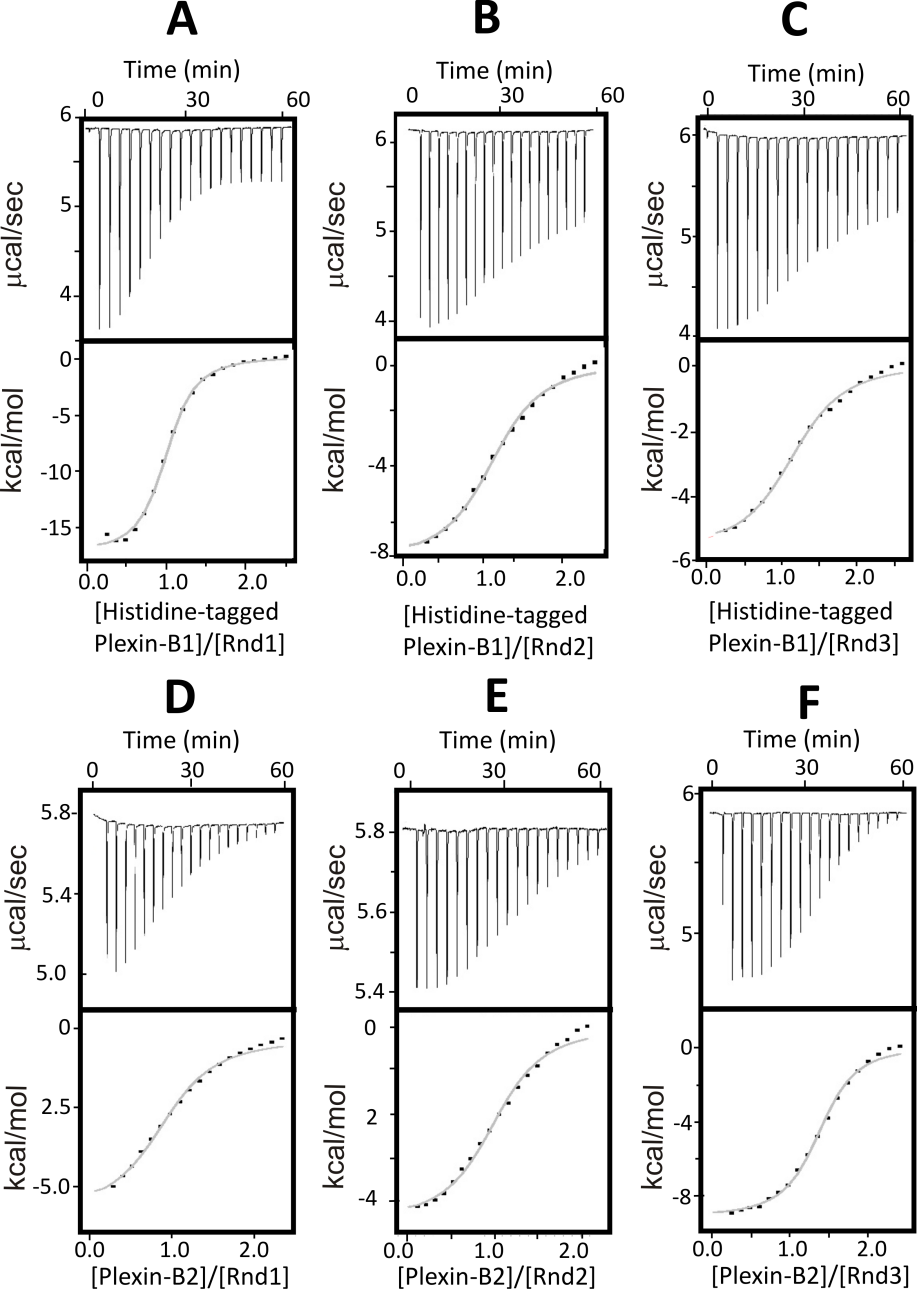
Analysis of the interaction of Plexin-B1-RBD and Plexin-B2-RBD with Rnd proteins. ITC experiments were conducted with buffer containing 150 mM NaCl. Raw titration data showing the thermal effect and normalised heats of interaction of injecting (A): Plexin-B1-RBD into Rnd1, (B): Plexin-B1-RBD into Rnd2, (C): Plexin-B1-RBD into Rnd3, (D): Plexin-B2-RBD into Rnd1, (E): Plexin-B2-RBD into Rnd2 and (F): Plexin-B2-RBD into Rnd3. The experiments were conducted in 50 mM sodium phosphate pH 7.0, 150 mM NaCl, 4 mM MgCl_2_ and 3 mM DTT at 25°C. The normalized heat of interaction was obtained in all cases by integrating the raw data and subtracting the heat of ligand dilution into the buffer alone. The grey line represents the best fit obtained by a non-linear least-squares procedure based on an independent binding sites model.

Often non-specific binding events arise from electrostatic interactions [31–33]. To minimise this, the buffer NaCl concentration in the ITC experiments was increased from 50 mM to 150 mM (Fig 1A and 1B). This resulted in a significant reduction in non-specific binding events, enabling the analysis of all the interactions tested. Our ITC measurements using 150 mM NaCl demonstrated a direct interaction between Plexin-B1 and all three Rnd proteins, revealing a monophasic 1:1 binding isotherm and a binding affinity in the low-micromolar range with dissociation constants (Kd) of 2.4, 7.6 and 6.6 μM for Rnd1, Rnd2 and Rnd3 respectively. The thermodynamics parameters are reported in Table 1.

**Table 1:** Thermodynamic parameters of the interaction between Rnd proteins and Plexin-B1-RBD or Plexin-B2-RBD.

Interaction	n	Kd (μM)	ΔH (kcal/mol)	-TΔS (kcal/mol)	ΔG (kcal/mol)
Rnd1/HisPlexin-B1	0.85 ± 0.01	2.37 ± 0.22	-15.56 ± 1.40	7.91 ± 1.36	-7.65 ± 0.09
Rnd2/HisPlexin-B1	1.16 ± 0.03	7.64 ± 2.45	-7.33 ± 0.74	0.3 ± 0.82	-7.03 ± 0.19
Rnd3/HisPlexin-B1	1.07 ± 0.04	6.62 ± 1.56	-9.73 ± 2.36	2.59 ± 2.39	-7.14 ± 0.08
Rnd1/Plexin-B2	1.05 ± 0.17	11.11 ± 1.98	-6.67 ± 1.56	-0.11± 1.67	-6.76 ± 0.12
Rnd2/Plexin-B2	1.15 ± 0.07	4.93 ± 0.84	-4.29 ± 0.23	-2.97 ± 0.29	-7.26 ± 0.10
Rnd3/Plexin-B2	1.23 ± 0.12	3.01 ± 0.28	-9.33 ± 0.11	1.80 ± 0.14	-7.53 ± 0.06

ITC experiments were carried out in 50 mM sodium phosphate pH 7.0, 150 mM NaCl, 4 mM MgCl_2_ and 3 mM DTT at 25°C. Values are the mean and standard deviation from fitted binding curves, obtained by a non-linear least-squares procedure based on an independent binding sites model.

### Analysis of Plexin-B2-RBD interactions by isothermal titration calorimetry

The interaction of Plexin-B2-RBD with each of the Rnd proteins was characterised by ITC, using buffer 2 (containing 150 mM NaCl) that yielded better results for the Plexin-B1 titrations (Fig 1C and 1D; Table 1). These associations generated well-interpolated sigmoid-shaped curves based on an independent and equivalent binding sites model centred on a 1:1 stoichiometry. These experiments revealed that Plexin-B2-RBD interacts with all 3 Rnd proteins. Under the experimental conditions used the binding affinity for Rnd1, Rnd2 and Rnd3 was found to be comparable but not identical, with Kd of 11.1, 4.9 and 3.0 μM respectively.

### Analysis of interaction stoichiometry by isothermal titration calorimetry and analytical gel filtration

Previous studies have shown that Plexin-B1-RBD may exist as dimer but that the dimeric form is disrupted by the interaction with GTPases [5]. Our ITC experiments indicate that Plexin-B-RBD and Plexin-B2-RBD interact with Rnd1/2/3 in a 1:1 molar ratio. Nonetheless, the previously reported homodimer of Plexin-B1 suggested that the complexes could consist of 1:1 (Plexin-B:Rnd) heterodimers or 2:2 (Plexin-B:Rnd) heterotetramers. Understanding the exact stoichiometry of these interactions is key, given a current working model suggesting that the inactive state of the intracellular region of plexin exists as a dimer through the RBD domain, and that binding to GTPases would disrupt the dimer, eliciting conformational changes responsible for plexin activation [5,25,34].

**Fig 2:**
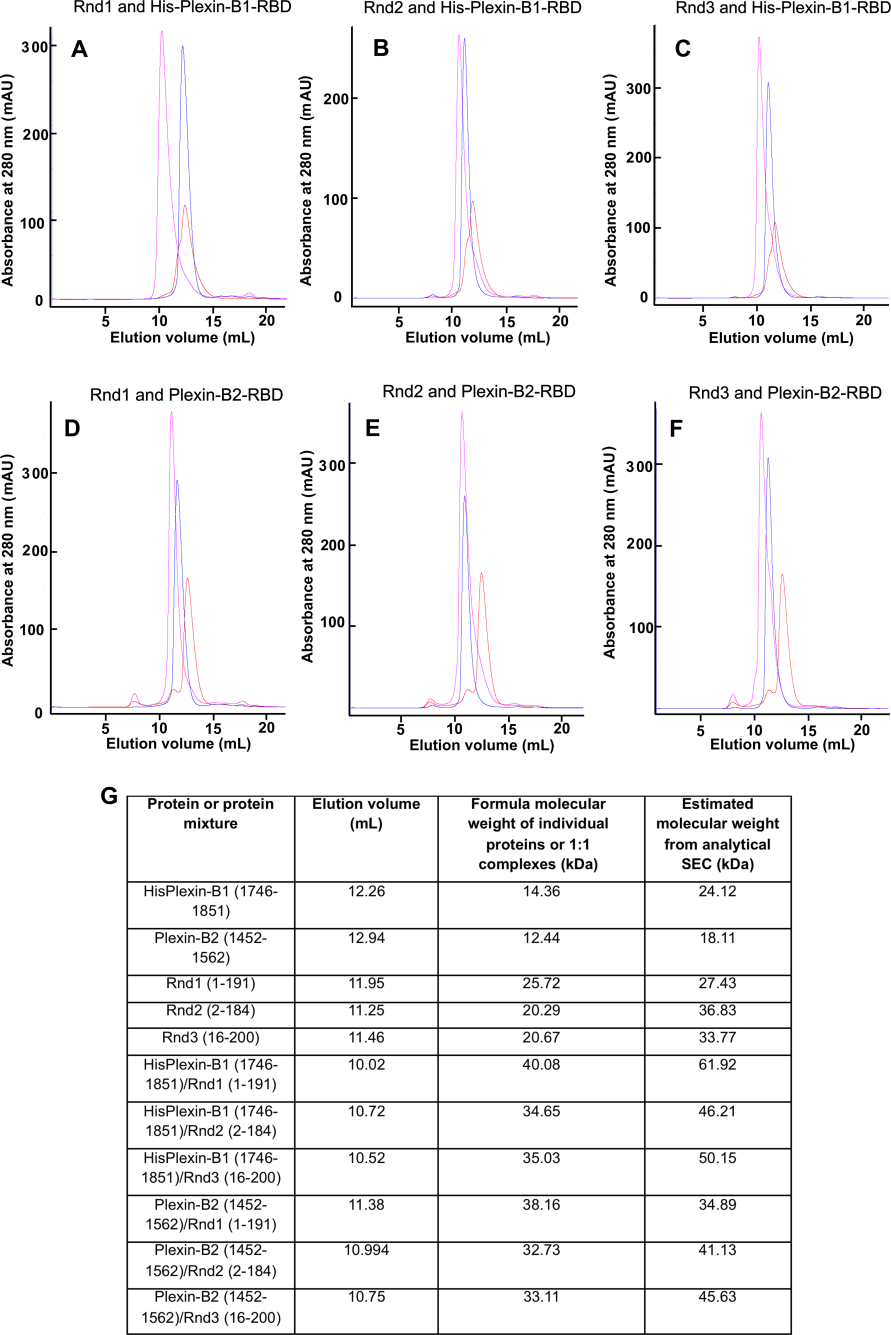
Size exclusion chromatography analysis of Rnd/Plexin complexes. UV traces from the elution of a 1:1 molar ratio mixture of Plexin-B1-RBD, Plexin-B2-RBD and each Rnd protein from a Superdex75 10/300 GL in 50 mM sodium phosphate pH 7.0, 150 mM NaCl, 4 mM MgCl_2_ and 3 mM DTT. The elution profile of the Rnd Protein-Plexin-B-RBD mixture were superimposed to traces of the single proteins injected alone for (A): Rnd1 and Plexin-B1-RBD, (B): Rnd2 and Plexin-B1-RBD, (C): Rnd3 and Plexin-B1-RBD, (D): Rnd1 and Plexin-B2-RBD, (E): Rnd2 and Plexin-B2-RBD and (F): Rnd3 and Plexin-B2-RBD. In all cases UV traces of complexes/mixtures are shown in pink, Rnd proteins in blue and Plexin-Bs in red. (G) Table showing the elution volumes, partition coefficients and formula and estimated molecular weights of proteins and complexes eluted.

We therefore performed analytical size exclusion chromatography (SEC). By estimating the size based on hydrodynamic radius of all the complexes compared with the isolated Plexin-B-RBD and Rnd proteins, these experiments showed that that all the complexes formed were 1:1 heterodimers (Fig 2).

### Mutagenic analysis of the binding site for Plexin-B1-RBD and Plexin-B2-RBD on Rnd3

**Fig 3:**
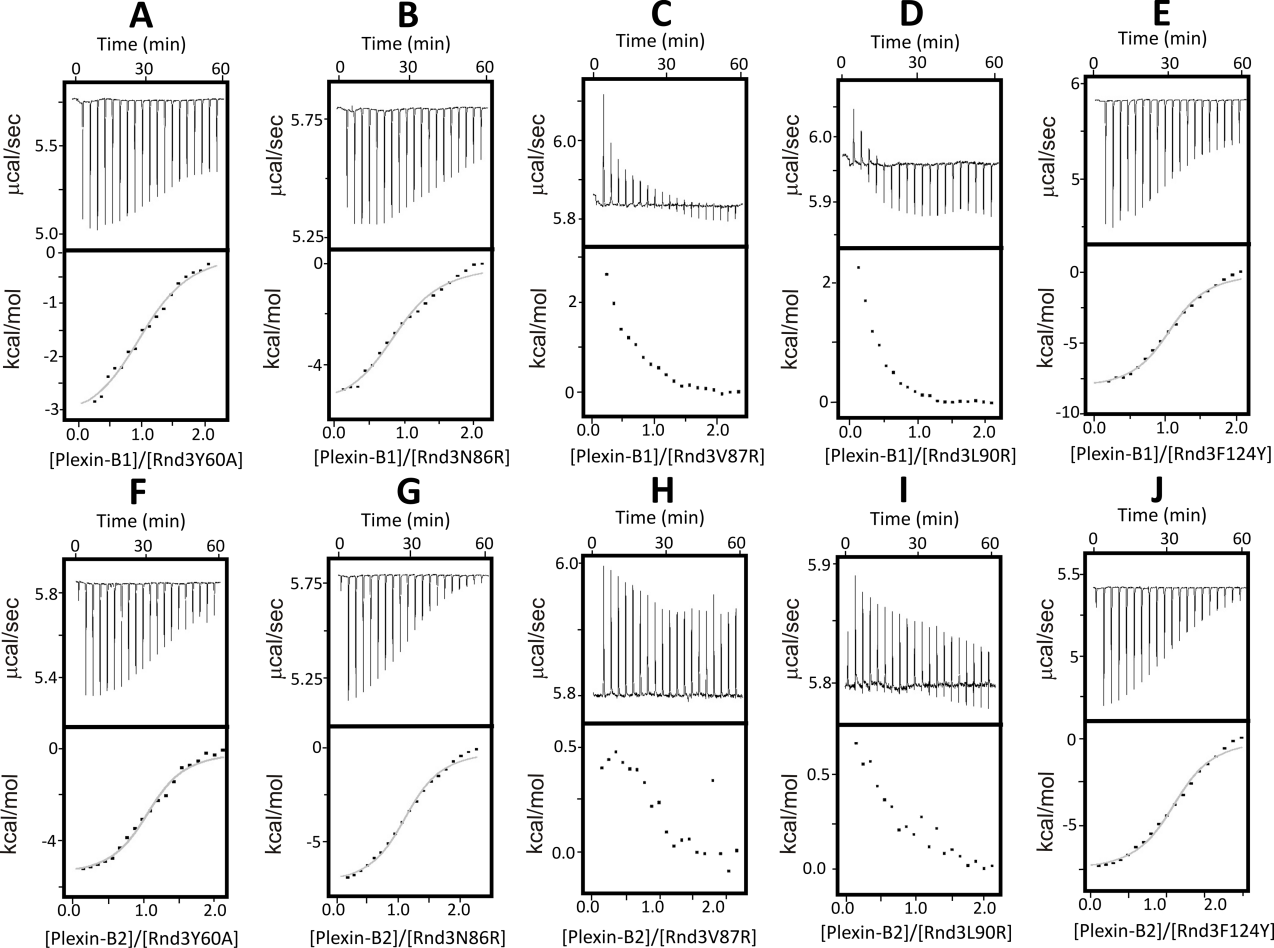
Analysis of the interaction of Plexin-B1-RBD and Plexin-B2-RBD with Rnd3 mutants. ITC raw titration data showing the thermal effect and normalised heats of interaction of injecting (A): Plexin-B1-RBD into Rnd3Y60A, (B): Plexin-B1-RBD into Rnd3N86R, (C) Plexin-B1-RBD into Rnd3V87R, (D): Plexin-B1-RBD into Rnd3L90R, (E): Plexin-B1-RBD into Rnd3F124Y, (F): Plexin-B2-RBD into Rnd3Y60A, (G): Plexin-B2-RBD into Rnd3N86R (H): Plexin-B2-RBD into Rnd3V87R, (I): Plexin-B2-RBD into Rnd3L90R and (J): Plexin-B2-RBD into Rnd3F124Y. The experiments were conducted in 50 mM sodium phosphate pH 7.0, 150 mM NaCl, 4 mM MgCl_2_ and 3 mM DTT at 25°C. The normalized heat of interaction was obtained in all cases by integrating the raw data and subtracting the heat of ligand dilution into the buffer alone. The grey line represents the best fit obtained by a non-linear least-squares procedure based on an independent binding sites model.

No structural information is currently available on Rnd3 complexes with Plexin-B RBDs. To understand better the binding interface of Rnd3 for the RBDs, we tested Rnd3 mutants for their ability to interact with both Plexin-B1 and B2 using ITC. Five point mutants of Rnd3, Y60A, N86R, V87R, L90R and F124Y [14], were subcloned into pETDuet-1 and purified as for wild-type Rnd3. The mutants had been designed based on superposing Rnd3 and Plexin-B2 onto the Rnd1-Plexin-B1 complex structure [14]. Two of these mutations, Rnd3-N86R and Rnd3-F124Y, did not have any effect on the ability of Rnd3 to interact with either Plexin-B1-RBD or Plexin-B2-RBD (Fig 3), consistent with co-immunoprecipitation studies carried out with Rnd3 mutants and the cytoplasmic domain of Plexin-B2 expressed in COS7 cells [14]. By contrast, the Rnd3 mutations V87R and L90R disrupted the interaction with Plexin-B RBDs, as no association could be observed by ITC in the experimental conditions used (Fig 3). The Rnd3-Y60A mutant displayed more complex differences from wild-type Rnd3: the dissociation constant for the interaction with both Plexin-B1-RBD and Plexin-B2-RBD did not change significantly compared to wild-type Rnd3, but the thermodynamics of the interaction (enthalpic versus entropic contribution) did, especially for the association with Plexin-B1-RBD (Table 2). As the thermodynamic signature of interaction can provide useful mechanistic clues, this suggests molecular differences in the way this mutant binds to Plexin-B1-RBD compared to wild-type.

**Table 2:** Thermodynamic parameters for the interactions of Rnd3 mutants with Plexin-B1 and B2, and comparison with co-immunoprecipitation experiments.

Interaction	n	Kd (μM)	ΔH (kcal/mol)	-TΔS (kcal/mol)	ΔG (kcal/mol)	Co-IP
Rnd3/Plexin-B2	1.23 ± 0.12	3.01 ± 0.28	-9.33 ± 0.11	1.80 ± 0.14	-7.53 ± 0.06	Yes
Rnd3Y60A/Plexin-B2	1.06 ± 0.02	3.06 ± 0.14	-5.64 ± 0.15	-1.88 ± 0.13	-7.51 ± 0.04	Weak
Rnd3N86R/Plexin-B2	1.13 ± 0.06	3.86 ± 0.69	-7.37 ± 0.42	-0.02 ± 0.52	-7.39 ± 0.11	Yes
Rnd3V87R/Plexin-B2	NB	NB	NB	NB	NB	No
Rnd3L90R/Plexin-B2	NB	NB	NB	NB	NB	No
Rnd3F124Y/Plexin-B2	1.14 ± 0.03	2.97± 0.33	-7.73 ± 0.32	0.18 ± 0.37	-7.54 ± 0.06	Yes
						
Rnd3/HisPlexin-B1	1.07 ± 0.04	6.62 ± 1.56	-9.73 ± 2.36	2.59 ± 2.39	-7.11 ± 0.08	No
Rnd3Y60A/HisPlexin-B1	1.02 ± 0.14	7.26 ± 2.04	-2.76 ± 0.50	-4.26 ± 0.65	-7.03 ± 0.14	ND
Rnd3N86R/HisPlexin-B1	0.91 ± 0.05	8.12 ± 0.95	-5.53 ± 0.32	-1.42 ± 0.30	-6.95 ± 0.07	ND
Rnd3V87R/HisPlexin-B1	NB	NB	NB	NB	NB	ND
Rnd3L90R/HisPlexin-B1	NB	NB	NB	NB	NB	ND
Rnd3F124Y/HisPlexin-B1	1.04 ± 0.08	4.60 ± 0.58	-8.65 ± 0.38	0.85 ± 0.80	-7.28 ± 0.08	ND

Thermodynamic parameters were measured by ITC for the interaction of each of the Rnd3 mutants with Plexin-B2-RBD (top rows) and Histidine-tagged Plexin-B1-RBD (bottom rows), measured in ITC buffer at 25°C. Values are the mean and standard deviation from fitted binding curves, obtained by a non-linear least-squares procedure based on an independent binding sites model. Co-immunoprecipitation (Co-IP) results are from [14] NB: no binding detected. ND: not determined.

These mutagenesis results did not highlight major differences in the interaction of Rnd3 with Plexin-B1-RBD and Plexin-B2-RBD (Table 2), suggesting similarities in the interaction surfaces in the two complexes, albeit this awaits confirmation from structural studies.

## Discussion

The cytoplasmic regions of Plexins interact with several small GTPases and hence it is important to determine whether different Plexins preferentially interact with distinct GTPases and to elucidate the molecular basis for any binding preferences. Here we focussed on the Rnd family of Rho GTPases, comprising Rnd1, Rnd2 and Rnd3, and their interaction with Plexin-B proteins. We carried out a systematic quantitative analysis comparing the binary association *in vitro* of the Rnd proteins with the RBDs of Plexin-B1 and Plexin-B2.

The interaction between Rnd1 and Plexin-B1-RBD has been studied previously, but not with PlexinB2-RBD. Here we find that PlexinB1-RBD interacts with Rnd1, Rnd2 and Rnd3 with comparable affinity, although the binding is stronger for Rnd1. Notably, ITC data reported previously [26] indicated a preferential association of PlexinB1-RBD for Rnd1 *versus* Rnd2. A possible explanation for this discrepancy may be due to the low salt buffer conditions used for the ITC experiments [26], and this may have prevented a full analysis of the ITC thermograms for the Plexin-B1:Rnd2 interaction because of non-specific association events (see above and S1 Fig). Our results are consistent with those found by Fansa et al. [6] and indicate that Plexin-B1 lacks a clear-cut binding specificity for the Rnd proteins on the basis of interaction with the RBD alone. Interestingly, co-immunoprecipitation analyses using full-length or the entire cytoplasmic domain of Plexin-B1 indicate that Rnd2 interacts much better than either Rnd1 or Rnd3 [14] (S1 Table), suggesting that regions outside of the RBD modulate Plexin-B1-Rnd interactions. In support to this view, ITC experiments performed in comparable experimental conditions revealed that the association of Rnd1 to the entire cytoplasmic domain of Plexin-B1 [35] was approximately 7-fold weaker than to the Plexin-B1-RBD alone [5]. This suggests that regions outside of the RBD weaken the interaction of Plexin-B1 with Rnd1, which could explain the apparent discrepancies between work using only the RBD and those using the cytoplasmic domain of Plexin-B proteins. Alternatively, the cytoplasmic domain of Plexin-B1 could normally exist in an auto-inhibited conformation, precluding interaction with the RBD. As yet unidentified post-translational modifications or interactions with other molecules could open up the conformation to allow full access to the RBD.

Similar to the Plexin-B1-RBD, we find that the Plexin-B2-RBD interacts with all the Rnd proteins, again with affinities in the same low micromolar range but with an order of preference Rnd3>Rnd2>Rnd1. This agrees with co-immunoprecipitation experiments in which full-length or the cytoplasmic domain of Plexin-B2 associated most strongly with Rnd3, followed by Rnd2 and then Rnd1 [14]. The interaction of Rnd3 with Plexin-B2 has been shown to contribute to Rnd3-induced cell shape changes and loss of actin stress fibres [14]. Unfortunately we were not able to obtain soluble recombinant Plexin-B3-RBD, so were unable to test its interactions with Rnd proteins. However, in co-immunoprecipitation experiments, Plexin-B3 showed a similar preference to Plexin-B1 for Rnd2 over Rnd1 or Rnd3 [14].

**Fig 4:**
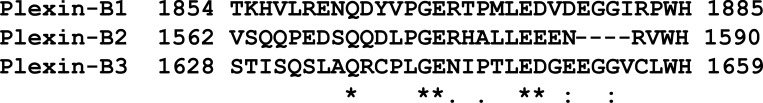
Sequence alignment of B1L region in Plexin-B proteins. Residues 1854–1885 of Plexin-B1, defined as B1L, were aligned with full length Plexin-B2 and Plexin-B3, using T-Coffee.

The small binding differences revealed by ITC between Rnd proteins and PlexinB RBDs appear unable to explain the different functional profile of these proteins. ITC experiments revealed overall similar affinities for all the protein tested, although in cells Rnd1 interaction with all plexin-B proteins was considerably weaker than that of Rnd2 [14]. Fansa et al. [6] suggested that a loop C-terminal to the RBD in PlexinB1, which they termed ‘B1L’, spanning residues 1854–1885, could play a significant role in determining binding specificity for Rnd proteins. A sequence alignment of this region performed using T-Coffee [36] (Fig 4) reveals some sequence variation with PlexinB2 and PlexinB3, perhaps supporting this hypothesis. In the future, an analysis of the contribution of these sequence variations to the interaction between Rnd proteins and Plexin-B-RBDs would be informative.

It is possible that the most important factor in determining the interaction of a specific Rnd protein with a Plexin-B protein in cells is determined by whether they co-localize in membranes. As previously suggested, while the apparent affinities measured of small GTPases for their targets are relatively weak, their shared membrane localisation facilitates their association in cells [37].

Taken together, it is likely that the RBDs of Plexin-B1 and Plexin-B2 are necessary but not sufficient to provide specificity for different Rnd proteins. In cells and in vivo, distinct Rnd proteins have been shown to be required for Plexin-B signalling [12,14,23,24]. The functional roles of the interactions between different Plexin-B and Rnd proteins may reflect in part relative levels of association with the RBDs as well as expression levels of each protein, their cellular localisations and contributions of other domains of each Plexin or other, as yet unidentified interacting proteins.

## Supporting information

S1 Fig Analysis of the interaction of Plexin-B1-RBD and Rnd1 and Rnd2 in low salt buffer. ITC raw titration data showing the thermal effect of injecting (A) Plexin-B1 into Rnd1 and (B) Plexin-B1 into Rnd2. The normalised heat of interaction was obtained by integrating the raw data and subtracting the heat of ligand dilution into the buffer alone. The red line represents the best fit curve, obtained by a non-linear least-squares procedure based on an independent binding sites model. The experiments were conducted in 50 mM sodium phosphate pH 7.0, 50 mM NaCl, 4 mM MgCl_2_ and 3 mM DTT at 25°C.
(TIF)

S1 Table Summary of previously published studies reporting on interactions between Plexin-B and Rnd proteins. For each study the primary citation, type of the interaction investigated, the technique used and experimental conditions used are reported. For ITC and SPR results, dissociation constant values (Kd) are reported.
(DOCX)

S2 Table Thermodynamic parameters for the interaction between Rnd1 and Plexin-B1-RBD in low salt conditions. Buffer used in this experiment was 50 mM sodium phosphate pH 7.0, 50 mM NaCl, 4 mM MgCl_2_ and 3 mM DTT at 25°C. Values are the mean and standard deviation from 5 fitted binding curves obtained by a non-linear least-squares procedure based on an independent binding sites model.
(DOCX)
