# Supplementary figures and images for: Analysis of the interaction of Plexin-B1 and Plexin-B2 with Rnd family proteins
Source: PLoS One. 2017 Oct 17;12(10):e0185899. doi: 10.1371/journal.pone.0185899 (PMC5645086; doi:10.1371/journal.pone.0185899)

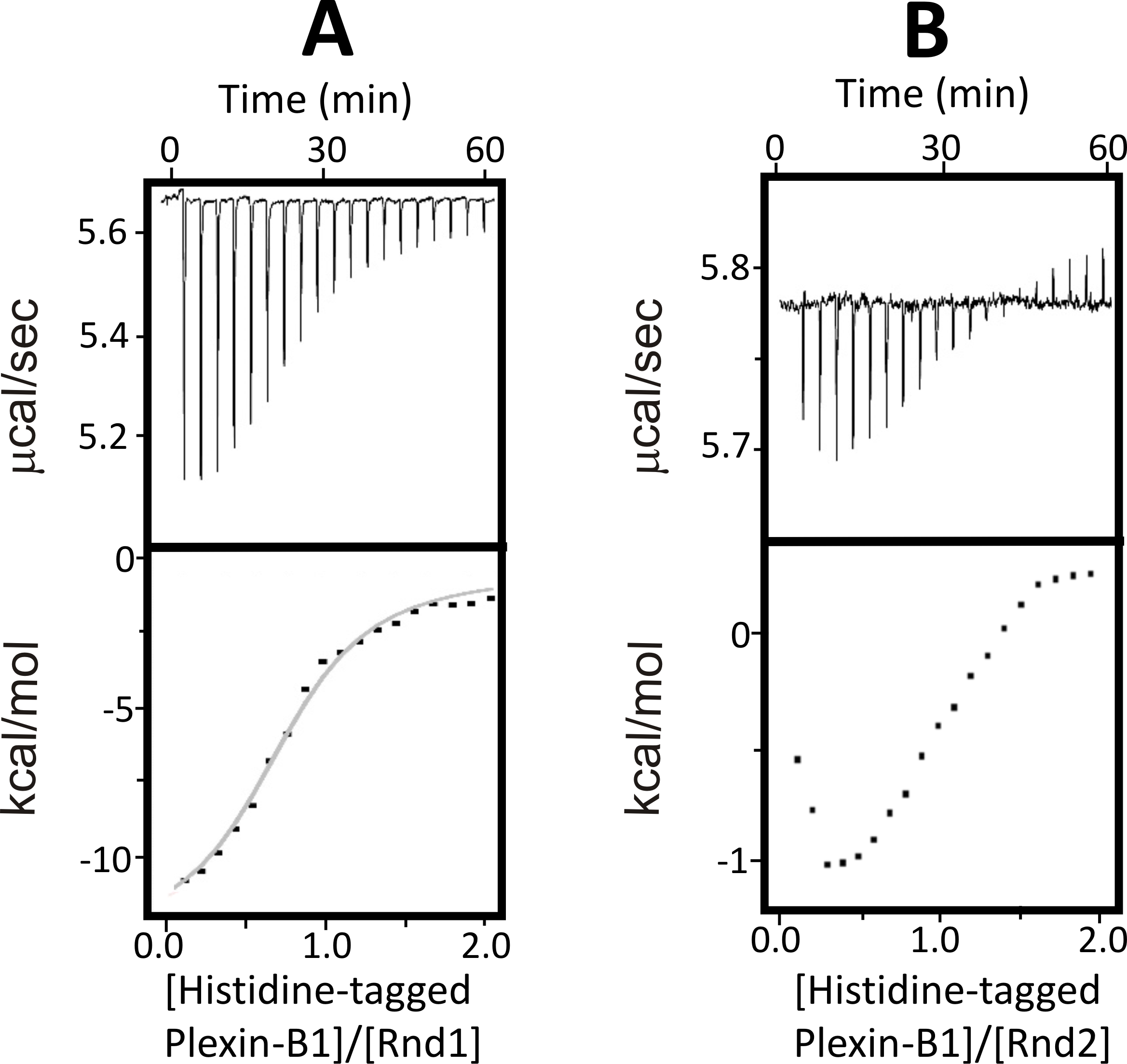

Supplement: S1 Fig — ITC raw titration data showing the thermal effect of injecting (A) Plexin-B1 into Rnd1 and (B) Plexin-B1 into Rnd2. The normalised heat of interaction was obtained by integrating the raw data and subtracting the heat of ligand dilution into the buffer alone. The red line represents the best fit curve, obtained by a non-linear least-squares procedure based on an independent binding sites model. The experiments were conducted in 50 mM sodium phosphate pH 7.0, 50 mM NaCl, 4 mM MgCl2 and 3 mM DTT at 25°C. (TIF) [file pone.0185899.s001.tif]
