# Supplementary material for: Analysis of the interaction of Plexin-B1 and Plexin-B2 with Rnd family proteins
Source: PLoS One. 2017 Oct 17;12(10):e0185899. doi: 10.1371/journal.pone.0185899 (PMC5645086; doi:10.1371/journal.pone.0185899)
Supplement: S1 Table — For each study the primary citation, type of the interaction investigated, the technique used and experimental conditions used are reported. For ITC and SPR results, dissociation constant values (Kd) are reported. (DOCX) [file pone.0185899.s002.docx]

| **Study** | Tong *et al., 2009 [*[*35*](#_ENREF_35)*]* | Hota & Buck*, 2009 [*[*34*](#_ENREF_34)*]* | Wang *et al*, 2011 [[26](#_ENREF_26)] | Wang *et al*, 2011 [[26](#_ENREF_26)] | Fansa *et al*, 2013 [[6](#_ENREF_6)] | McColl *et al*. 2016 [[14](#_ENREF_14)] |
| --- | --- | --- | --- | --- | --- | --- |
| Technique | ITC | ITC | ITC | SPR | ITC | Co-IP |
| Buffer Used | 20 mM Tris pH 7.5, 1 mM TCEP, 50 mM NaCl, 4 mM MgCl_2_ | Several | 50 mM sodium phosphate pH 7.0, 50 mM NaCl, 4 mM MgCl_2_ and 0.5 mM TCEP | 10 mM HEPES pH 7, 150 mM NaCl, 4 mM CaCl_2_, 4 mM DTT, 0.005% (vol/vol) surfactant p20. | 30 mM Tris/HCl pH 7.5, 50 mM NaCl | Cellular Assay |
| Plexin-B1 Fragment Used | Cytoplasmic domain (1511-2135) | RBD (1742-1862) | RBD (1746-1852) | RBD (1746-1852) | RBD+’B1L’ (1724–1903) | Cytoplasmic domain and full length (exact residues not stated) |
| Plexin-B2 Fragment Used | Not Tested | Not Tested | Not Tested | Not Tested | Not Tested | Cytoplasmic domain and full length (exact residues not stated) |
| Measure of Interaction | Kd | Kd | Kd | Kd | Kd | Co-IP |
| Plexin-B1-Rnd1 | 35 μM | In the 6-10 μM range | 5.5 μM | 1.51 μM | 3.18 μM | Very weak |
| Plexin-B1-Rnd2 | Not Tested | Not Tested | >20 μM | 1.39 μM | 4.56 μM | Strong |
| Plexin-B1-Rnd3 | Not Tested | Not Tested | Not Tested | Not Tested | 8.54 μM | Very weak |
| Plexin-B2-Rnd1 | Not Tested | Not Tested | Not Tested | Not Tested | Not Tested | Very weak |
| Plexin-B2-Rnd2 | Not Tested | Not Tested | Not Tested | Not Tested | Not Tested | Strong |
| Plexin-B2-Rnd3 | Not Tested | Not Tested | Not Tested | Not Tested | Not Tested | Strong |

S1 Table
