# Supplementary material for: Analysis of the interaction of Plexin-B1 and Plexin-B2 with Rnd family proteins
Source: PLoS One. 2017 Oct 17;12(10):e0185899. doi: 10.1371/journal.pone.0185899 (PMC5645086; doi:10.1371/journal.pone.0185899)
Supplement: S2 Table — Buffer used in this experiment was 50 mM sodium phosphate pH 7.0, 50 mM NaCl, 4 mM MgCl2 and 3 mM DTT at 25°C. Values are the mean and standard deviation from 5 fitted binding curves obtained by a non-linear least-squares procedure based on an independent binding sites model. (DOCX) [file pone.0185899.s003.docx]

| **Interaction** | **n** | **Kd** **(μM)** | **ΔH** **(kcal/mol)** | **-TΔS** **(kcal/mol)** | **ΔG** **(kcal/mol)** |
| --- | --- | --- | --- | --- | --- |
| Rnd1/HisPlexin-B1 | 0.94 ± 0.17 | 5.37 ± 1.75 | -16.24 ± 4.70 | 9.02 ± 4.77 | -7.22 ± 0.19 |

S2 Table
